# Non-Destructive Soluble Solids Content Determination for ‘Rocha’ Pear Based on VIS-SWNIR Spectroscopy under ‘Real World’ Sorting Facility Conditions

**DOI:** 10.3390/s19235165

**Published:** 2019-11-26

**Authors:** Dário Passos, Daniela Rodrigues, Ana Margarida Cavaco, Maria Dulce Antunes, Rui Guerra

**Affiliations:** 1 CEOT, Universidade do Algarve, Campus de Gambelas, FCT Ed.2, 8005-189 Faro, Portugal; danielaac_rodrigues@sapo.pt (D.R.); acavaco@ualg.pt (A.M.C.); 2 MeditBio, Universidade do Algarve, Campus de Gambelas, FCT Ed.8, 8005-189 Faro, Portugal; mantunes@ualg.pt

**Keywords:** VIS-SWNIR spectroscopy, diffuse reflectance, soluble solids content, machine learning, fruit’s internal quality, non-destructive measurements

## Abstract

In this paper we report a method to determine the soluble solids content (SSC) of ‘Rocha’ pear (*Pyrus communis* L. cv. Rocha) based on their short-wave NIR reflectance spectra (500–1100 nm) measured in conditions similar to those found in packinghouse fruit sorting facilities. We obtained 3300 reflectance spectra from pears acquired from different lots, producers and with diverse storage times and ripening stages. The macroscopic properties of the pears, such as size, temperature and SSC were measured under controlled laboratory conditions. For the spectral analysis, we implemented a computational pipeline that incorporates multiple pre-processing techniques including a feature selection procedure, various multivariate regression models and three different validation strategies. This benchmark allowed us to find the best model/preproccesing procedure for SSC prediction from our data. From the several calibration models tested, we have found that Support Vector Machines provides the best predictions metrics with an RMSEP of around 0.82 ${}^{\circ }$Brix and 1.09 ${}^{\circ }$Brix for internal and external validation strategies respectively. The latter validation was implemented to assess the prediction accuracy of this calibration method under more ‘real world-like’ conditions. We also show that incorporating information about the fruit temperature and size to the calibration models improves SSC predictability. Our results indicate that the methodology presented here could be implemented in existing packinghouse facilities for single fruit SSC characterization.

## 1. Introduction

Technology assimilation into the agricultural field has led to big leaps in productivity and management capacity. The concept of Agriculture 4.0 [1,2] is therefore a natural path that will lead to even more progresses. Quality control of fresh fruits is an area that is transversely important, spreading from producers, retailers and ultimately impacting the consumer. The development of affordable and more reliable methods of controlling food quality and managing crops is imperative in order to maximize productivity and profitability and to minimize environmental impacts.

For several years, visible and near infrared (VIS-NIR) spectroscopy has contributed to this trend by providing non-destructive methods of probing the internal quality of fresh fruits, vegetables, cereals and other edibles [3,4,5,6,7,8]. In reflectance VIS-NIR spectroscopy, a fruit for example is irradiated with VIS-NIR light and the consequent reflected radiation is measured. Part of the incident radiation penetrates the fruit and scatters around interacting with the chemical compounds in its interior. The portion of radiation that finds its way out of the fruit’s tissue carries a spectral signature of the chemical molecules that absorbed certain wavelengths. For simple structure materials, these signatures are usually visible as well defined peaks in an absorption spectrum. However, for biological tissues, the detection of individual absorption peaks in this spectral range is hard because these peaks tend to overlap and spectra are dominated by the water spectrum which masks other chemical signatures under its broad peaks [3]. Because of this, in order to retrieve information from this spectral window, a multivariate analysis approach is usually required. A particularly interesting problem that this technology is trying to solve, is the ability of quantify directly the Soluble Solids Content (SSC) in fruits. This is relevant because the SSC level comprises circa 85% sugars (sucrose, fructose and glucose) in most fruits (with the exception of certain citrines), and is accepted in general, as a good indicator of fruit sweetness [9]. The ultimate goal is to offer the producers and consumers reliable source of information about the flavour of fruits.

Pears are one of the most popular fruit worldwide and very appreciated in large economical markets such as China. Due to its potential commercial importance, the target fruit of this study is the ‘Rocha’ pear variety (*Pyrus communis* L.), a protected designation of origin (PDO) fruit and one of the most cultivated fruit crops in Portugal with a net production of 202 thousand tones in 2017 and moving an economy of around 95 million euros [10] in this country. We notice that, although this work focus specifically on the non-destructive quantification of SSC in the ‘Rocha’ pear, the techniques described herein could be generalized to other thin peel fruits (e.g., apples, plums, etc).

The vast majority (if not all) of the works about VIS-NIR prediction of SSC in pears indicate that this method is able to extract relevant SSC information from this spectral range [11,12,13,14,15,16]. However many of the methodologies/models proposed in the literature do not work well in real world conditions. By ‘real work conditions’ we mean non-contact spectral measurements under the conditions that can be found presently inside commercial inline sorting facilities already operating in packinghouses (large conveyor belt structures working inside warehouses). In terms of the practical implementation of several literature models we can highlight two major drawbacks. The first is related to the very controlled laboratory conditions in which these studies are done (temperature, measuring distances, dark room, etc.) and many times using expensive/custom state of the art equipment. Multiple temperature levels of samples in particular are rarely used in these works despite temperature being the external factor that most influences the spectrum [17,18]. Temperature influences the strength of molecular bonds and their vibrational modes which result in a shifting and broadening of absorption bands. O-H bonds, that are present in water and sugar molecules are particularly sensible to this effect. Due to these facts, temperature induced spectral changes difficult the inference of multivariate methods and tend to decrease models performance [19,20,21]. The second drawback is related to the small number of samples usually used in these works. Most studies rely on a small number of samples that range from a few tens to a couple hundred at maximum and usually in the form of very uniform sets of fruits (same origin, shelf lifetime, harvest season, maturity level, etc.). Although the published results are a good prof-of-concept, we believe that most of these data sets do not account for the existing wide range of biological variability present in fruits. Under such tight conditions, models tend to give results that we find over-optimistic and very hard to reproduce for practical implementations. Moreover, complex models have been shown to yield better predictability performance using homogeneous validation strategies (sample randomization and train-test split) but it is not clear if the advantage is kept for an heterogeneous validation procedure where validation is performed on truly independent sets (different orchard/year/producer) and not a subset of the whole universe of samples obtained by homogeneous sampling [22,23]. Another aspect that is seldom discussed in the literature is the fact that model performance should be limited by instrument and sampling uncertainties.

In this study we try to overcome some of these short-comings by implementing an experimental design that accounts for most of the mentioned issues. Our methodology relies on rapid spectra acquisition times using off-the-shelf affordable detectors (spectral range 470–1150 nm range) and common optical components; it simulates some of the constraints found in inline sorting facilities (e.g., variable distance between fruit surface and detector, lighting conditions, etc.); it uses a large data set comprising 3300 samples (one order of magnitude larger than those used in the previously cited works) for model optimization that takes into account a large biological variability (pears from multiple producers, orchards, with a wide range of storing times and conditions, sizes, harvest seasons and ripening stages); and finally we take into account the role of temperature to simulate the thermal conditions that the pears are subjected during the harvesting season and their subsequent storage in climatized/atmosphere controlled chambers. The final goal of this work is to find what combination of spectral data pre-processing and model, is able to yield the best SSC prediction results, when applied to a set of ‘Rocha’ pears measured in conditions similar to those found in inline sorting facilities. For that, we implemented a data analysis pipeline that incorporates different validations strategies (homogeneous and heterogeneous), multiple data pre-processing methods and 4 multivariate models (including model optimization procedures). In the following section we detail each step of this benchmark.

## 2. Materials and Methods

### 2.1. Target Fruit

**Figure 1:**
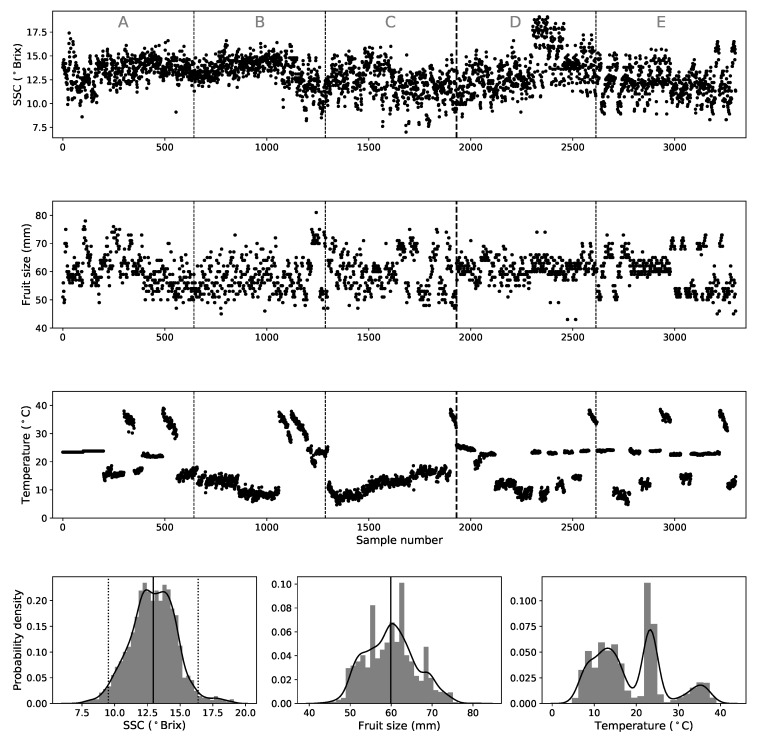
(**Top**): Macroscopic fruit attributes (SSC, fruit size and peel temperature) for the 3300 samples. The *External Validation* subsets are separated by vertical dashed lines and marked with letters. Fruits from A, B and C belong to the 2010 harvest season while those from D and E belong to the 2011 harvest season. The thick vertical dashed line marks the separation. (**Bottom**): The solid curves represent the statistical distributions, given by kernel density estimation (KDE) plots of the macroscopic attributes. The gray bars in the background represent the normalized histogram of the data. The solid vertical lines represent mean values and the dotted ones in the SSC panel enclosure 95% of the samples (±2σ).

The harvesting season of the ‘Rocha’ pear in Portugal happens during summer and extends from the middle of August to the middle of September. These fruits undergo temperatures that can go from as high as 40 ${}^{\circ }$C during harvest to the −0.5 ${}^{\circ }$C of cooling chambers. The fruits used in this study were acquired during 2011 from local stores and came originally from different orchards and producers from the center western region of Portugal. The experiment was done over two time periods, the first spanning from March to the end of May 2011 (measurements number 0 to 1930) and the second spanning from middle of August to the end of November 2011 (measurements 1931 to 3300). See Figure 1. The pears used in the first acquisition period correspond to fruits harvested in the summer of 2010 and kept under low temperature and controlled atmosphere chambers that allow them to be stored and shipped throughout the whole year. The fruit from the second period belong to the 2011 harvest season and represent fresh fruits that did not went through extensive chamber storage periods. This option of sampling fruits throughout the year allowed us to have pears with multiple maturation states in out data set.

### 2.2. Experimental Setup and Methodology

The pears were acquired from local markets in batches of approximately 50 and each fruit was sampled individually within a few days. This procedure was repeated over many weeks. For each fruit we collected two spectra and two SSC measurements (180 degrees apart, along the fruit’s equator). The spectra were collected in an experimental setup designed to mimic the ‘real world’ conditions mentioned in the introduction. Two multimode optical fibers, with a core of 600 μm and a NA = 0.22, coupled to collimating optics, were used for illuminating and collecting light from the fruits. The illumination fiber was bifurcated and the input end was connected to a white light halogen source (Ocean Optics HL-2000-FHSA, Ocean Optics, Largo, FL, USA). The two exit ends were mounted 5 cm apart, making a 5 degree angle with the vertical with the two collimated beams of light converging. The collection fiber was connected to an Hamamatsu TG-9405CA (Hamamatsu, Shizuoka, Japan) spectrometer (with 5 nm spectral resolution) where the spectrum between 432 and 1147 nm was recorded. The acquisition time for each pear was 50 ms. This fiber was positioned between the two illumination fibers (in the same plane) with a 0 degree angle with the vertical aimed at the region between the illumination spots. The collection/illumination fibers were located 170 mm above the cup that held the fruit and the spectra were acquired from the pear’s equatorial region. Therefore, in this setup, the distance between the illumination/collection fibers and the fruit’s surface was variable (due to the pears’ different sizes). For this same reason, the distance between the two illuminated regions at the fruits surface was also variable, i.e., the sub-surface scattering path differs between fruits with different equatorial radii. Spectra from very small fruits (with diameter < 42 mm) were removed due to specular light contamination. The white light reference spectrum was obtained using a white teflon disc and, prior to every measurement, a dark spectrum was also obtained with the light source turned off. All measurements (reference included) were done in a dimly lit room (not completely dark). As usual, the reflectance spectrum *R*, was computed by subtracting the dark spectrum from the sample’s raw spectrum and dividing this by the reference spectrum (dark corrected as well).

Each of the acquired 3300 spectra has 1024 points distributed between 432 nm and 1147 nm. In terms of modelling, the 1024 wavelengths (i.e., the spectral amplitude at each wavelength) is what we refer individually to as “*features*” or collectively as the “*X-block*”. Note that the two spectra per fruit were not averaged together, they were used individually. The measurements span across three different temperature ranges. Samples were either measured at room temperature or after being stored on a cooling or heating chamber over several hours before acquiring the spectrum allowing us to mimic the broad range of temperatures that fruits withstand during the harvesting and storage periods. In our case this range varies from 4.7 ${}^{\circ }$C to 39 ${}^{\circ }$C. Before each spectral measurement the local peel temperature was registered using an infrared thermometer (Fluke FoodPro Plus, Everett, WA, USA).

After the spectra were collected, the same probed equatorial regions of the pears were cut out, squeezed and the SSC (in ${}^{\circ }$Brix) was measured using a digital refractometer (Atago Model PAL-1, Atago Co. LTD, Tokyo, Japan). We note that this is the same procedure that the majority of producers/retailers use to measure the SSC of crops at room temperature (20–25 ${}^{\circ }$C) and set its market value. The SSC content of the pears in this study varies from 7 to 19.1 ${}^{\circ }$Brix with a mean value of 13 (nearly 95% of the pears have SSC between 9.5 and 16.4 ${}^{\circ }$Brix). In this work we also refer to the SSC as “*Y-block*” in terms of modelling. The equatorial diameter of every sample was also registered. In our data set, fruit size spans from 43 to 81 mm and has mean value of 60 mm. Figure 1 shows the macroscopic parameters (SSC, temperature and size) and their statistical distributions.

### 2.3. Variability Associated with the Standard Destructive Measurements of SSC

**Figure 2:**
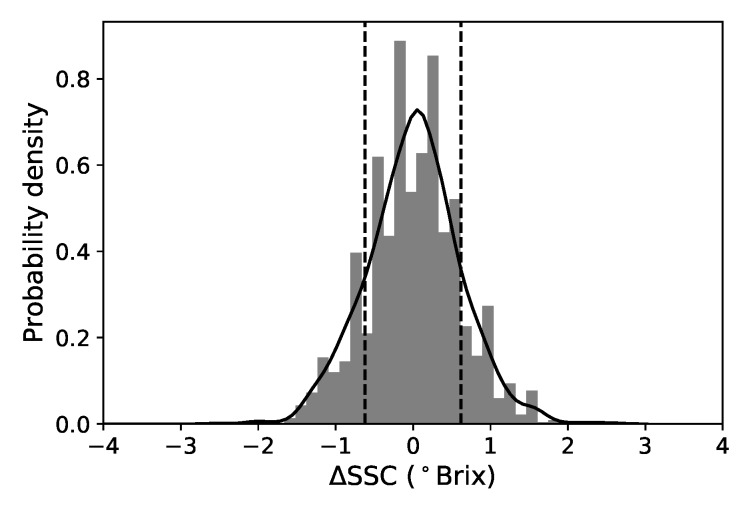
Normalized histogram and KDE plot of the SSC variation for all samples. The mean value is approximately 0 and the standard deviation is 0.62 ${}^{\circ }$Brix (represented by the vertical dotted lines).

Since we have two SSC destructive measurements for each fruit, we can take advantage of this and extract some information of how this quantity varies per pear. We subtract the two values and define ∆SSC as the SSC variation “per fruit” (see Figure 2). Additional laboratory tests, where repetitive SSC measurements were done in 8 different regions of individual pears, over several tens of fruits (Cruz et al. unpublished), indicate that this variability is associated with the experimental error in the SSC measurement with the refractometer and due to the non-uniform distribution of soluble solids throughout the fruit. We note that in a sorting setup, a single SSC value is assigned to a fruit and therefore it is useful to have in mind that there is always an intrinsic uncertainty associated with this value (even in controlled laboratory conditions). This information is important because it allows us to set realistic expectations about the implemented model’s predictability. We can roughly interpret the standard deviation of this distribution (0.62 ${}^{\circ }$Brix) as a lower limit of prediction for single fruits for all models. The root mean squared difference between both sides of the pears is 0.47 ${}^{\circ }$Brix and the two values do not coincide because the distribution is not exactly normal. Reference [11] reported a slighter lower value of 0.4 ${}^{\circ }$Brix but measured for a much smaller sample and much narrower SSC distribution.

### 2.4. Models and Pre-Processing Techniques

Our analysis pipeline incorporates 4 multivariate regression models and several pre-processing techniques. We tested two calibration models already applied to pears spectra for SSC quantification: Partial Least Squares (PLS) and Multiple Linear Regression (MLR) [11,12,13,14,15,24], another model that is gaining traction in Chemometrics and that has been applied in similar contexts, Support Vector Machines (SVM) [25,26] and a simple Multi Layer Perceptron (MLP) neural network for comparison purposes [27,28,29]. The numerical analysis of the data was done using Python 3.6. For PLS, MLR and SVM models we used the Scikit-Learn package [30] and to build the MLP neural network model we used TensorFlow 1.2 running on an NVidia GTX 1070 Ti GPU.

**Figure 3:**
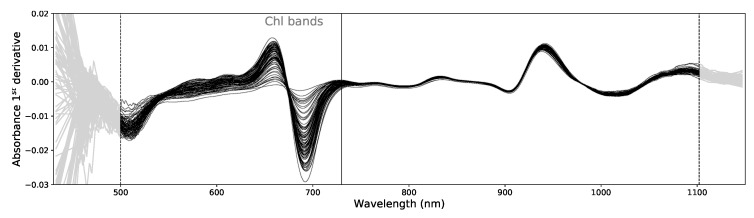
Example of 100 absorbance 1st derivative spectra. The gray data points at both ends of the spectra were not used. The vertical solid line marks the no Chlorophyll spectral data (noChl) that excludes the Chl bands below 730 nm.

For this kind of problem, several pre-processing procedures have been suggested in the literature [3,8,31]. These techniques usually enable better data uniformization, systematic errors correction and improvement of signal-to-noise levels. Following the most common practices in the field, we tested several pre-processing procedures with the objective to find out which one performs best as SSC predictor for our data. We first computed the absorbance spectra, log(1/R), from the original reflectance spectra, *R* and afterwards (using the full spectral range), we computed the absorbance’s 1st and 2nd (smoothed) derivatives using the Savitzky-Golay algorithm [32] with a window of 51 points and a polynomial degree of 2. After this step we trimmed 75 data points at the beginning and at the end of the spectra (see Figure 3) to remove smoothing windows effects and the noisier regions of the detector. Following this step we applied the Standard Normal Variate (SNV) correction [33] to the absorption, 1st and 2nd derivatives spectra, yielding a total of 6 pre-processed data types (the original absorbance spectra, the 1st and 2nd derivatives and their SNV counterparts).

Besides using the available spectral data, we also used the temperature and fruit size information by appending these two attributes as extra features (in the *X-block*) to our data sets (after the spectral pre-processing). Reference [18] reported that the collinearity between the spectrum regions that are affected by temperature and the temperature measurements could lead to regression models that capture and correct for the effect of temperature variations. These authors also briefly mentioned that non-linear corrections to temperature might be useful for some multivariate methods. In future works we intend to explore this option. As a result of adding this extra information to our data sets we end up with 12 pre-processed data types: 3 spectra (absorbance and its two derivatives), 3 spectra SNV corrected, plus a version of the 6 previous extended with temperature and fruit size.

We also took into account the effect of Chlorophyll (Chl) present in the fruit’s peel. Chl absorption (around 680 nm) is closely correlated with SSC, but, this correlation does not imply a causal effect. As pears gets more ripe, Chl levels in the peel decrease. At the same time physiological and biochemical processes in the fruit’s pulp also evolve in the direction of increasing the SSC. Indeed, it is very common to find a freshly harvested fruit with a high SSC and a much riper one with a lower value. It all depends on the initial base SSC level of the fruit. Due to its large variation during maturation, the Chl peak is a strong feature that models tend to capture instead of sugar specific bands that are less evident. O-H and C-H bonds in sucrose, fructose and glucose, have multiple signatures at 730–780 nm, 840 nm, 910–930 nm and at 960–980 nm due to a combination of third and fourth vibrational overtones (stretching and bending) and at 1100 nm due to C-H/C-H2 combination bands [34]. These feature tend to be masked by the water spectrum which makes capturing their variations more difficult. Based on these facts we find useful to test our models not only by using the full spectra, but also by excluding the Chl bands associated with the ripening process. All those pre-processed data types mentioned earlier were also tested excluding the Chl absorption bands below 730 nm.

### 2.5. Validation Strategies and Data Sets

In this analysis we implemented 3 different validation strategies, for which we reshaped the data into different data sets. The first strategy follows the common procedure of shuffling/randomizing the samples and then separate them into calibration (80%) and validation (20%) subsets. In some of the literature this strategy is called *Internal Validation* (*IV*) and creates an homogeneous data set. We applied this procedure to what we call “*Big*” data set that contains the full 3300 samples available.

**Figure 4:**
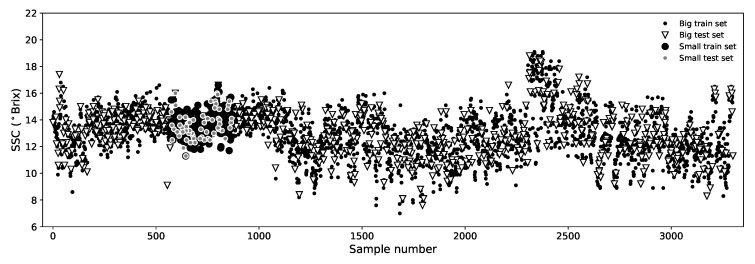
Distribution of calibration/validation samples in both *Big* and *Small* data sets after the shuffling process.

Also under an *IV* strategy, we defined a smaller data set that is restricted to 300 samples only and represents a much more uniform set of pears, all measured in the same week and having a much more uniform temperature and size ranges. This set was also shuffled and divided into calibration (80%) and validation (20%) subsets and we named it “*Small*” data set (see Figure 4). We use the *Small* data set for comparison purposes with most of the literature because a great deal of works rely on very uniform data sets of similar size. This also allows us to have a notion of the impact that the size and uniformity of the data set has in the model performance.

The third strategy is what we call *External Validation* (*EV*). As mentioned before, the fruits used in this work came from two different harvesting seasons (2010 and 2011). Therefore we decided that it would be interesting to explore this embedded chronological information [22] by creating an heterogenous data set. We divided the data into 5 subsets ordered chronologically and labeled as A, B, C, D, and E (see top panel of Figure 1). Then we applied the following strategy, use the aggregated ABCD for calibration and E as validation subset. We did this for the 5 possible ABCDE combinations. In doing so we are effectively using data across different storage conditions and different harvests for the calibration models. We believe that the average of these predictions gives us a more robust measure of the predictive capability of a calibration model because calibration and validation samples do not come from the same universal set (one that results from a randomized sample partition). A detailed investigation of the difference between internal and external validations in ‘Newhall’ oranges (*Citrus sinensis* L. Osbeck), as well as a more detailed explanation of the procedure can be found in [35].

**Figure 5:**
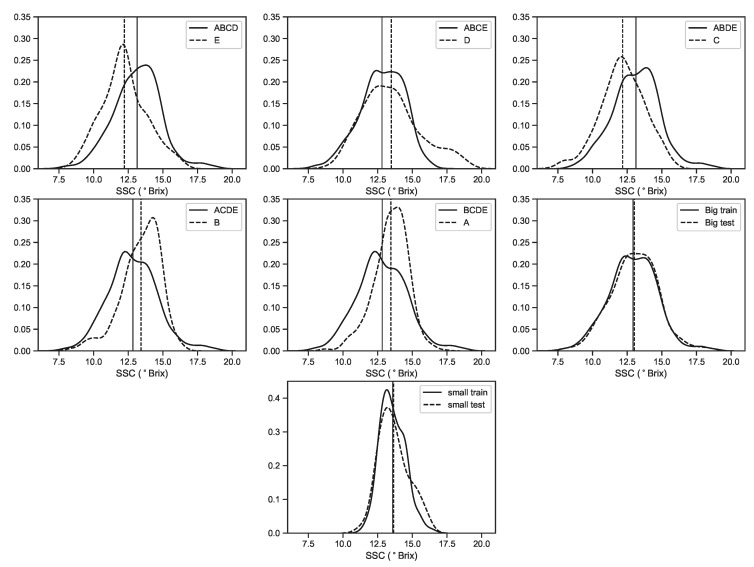
KDE plots of SSC for all data sets. The vertical axis represent the probability density. The solid curves represent the distributions of SSC values used for calibration and the dashed lines indicate the distributions of SSC values used for validation/predictions. The vertical thin solid/dashed lines represent the mean of the calibration/validation distributions respectively.

We can see from Figure 5 that in the *EV* subsets, there are clear differences between calibration and validation data sets in terms of statistical distributions. This will help assess how good the models are in generalizing the predictions when applied to sets of fruits with a different properties. For the *Big* and *Small* data sets, this is not an issue, because due to the shuffling/randomization of data before the splitting of the sets, much more homogeneous distributions are created.

### 2.6. Performance Metrics and Multivariate Model Optimization

In what follows we present the optimization methods used for the PLS, MLR, SVM and MLP models(As a side note, we would like to mention that our analysis pipeline was designed to optimize models for the *Big* data set because *Internal Validation* is much more widely used in the literature. As we will discuss later, this led to some problems when applying it to the *Small* data set due to the number of samples to features ratio.). Each of theses models have been hailed multiple times in the literature as highly efficient for predictions based on Chemometrics and several Spectroscopic applications. As usual, the validation sets were only used for computing the prediction metrics and were completely excluded from the models’ calibration/optimization procedures. The performance metrics we rely are the Root Mean Square Error of Cross-Validation (RMSECV), Root Mean Square Error of Calibration (RMSEC), the Root Mean Squared Error of Prediction (RMSEP), the Squared Correlation Coefficient (R${}^{2}$), the Coefficient of Variation (%) = (RMSEP/mean(y${}_{val}$)) × 100 and the Prediction Gain (PG) = (RMSEP if prediction = mean(y${}_{cal}$))/RMSEP. The pre-processing data type that has the lowest RMSEP is what we choose as the best predictor. RMSEP and RMSEC are always presented in ${}^{\circ }$Brix for a more intuitive error evaluation.

The coefficient of variation gives a measure of the prediction error relatively to the order of magnitude of the values to predict. The prediction gain compares the RMSEP obtained by the simplest possible prediction by using a “zero-order model” with the actual RMSEP obtained by the model under test. The zero-order model corresponds to predicting always the same value for all validation samples, this value being the calibration mean. The ratio of the two RMSEP values thus gives the number of times the tested model is better than the zero-order model. For example, a PG = 2 means that the RMSEP obtained through the tested model is half the one obtained through the zero-order model. The Prediction Gain generalizes the usually adopted Standard Deviation Ratio (SDR = std($y_{val}$)/RMSEP), but is a more fair statistic than SDR. Indeed, it is easy to see, from the mathematical expression of the standard deviation, that std($y_{val}$) = (RMSEP if prediction = mean(y${}_{val}$)). Thus

(1) $$\begin{array}{ccc} SDR & = & \frac{\mathrm{RMSEP}\;\mathrm{if}y_{pred,i}=mean\left(y_{val}\right),\;\forall i}{RMSEP} \end{array}$$

(2) $$\begin{array}{ccc} PG & = & \frac{\mathrm{RMSEP}\;\mathrm{if}y_{pred,i}=mean\left(y_{cal}\right),\;\forall i}{RMSEP} \end{array}$$

Therefore, in SDR there is the implicit assumption of the knowledge of the validation mean, which is actually unavailable, while in PG there is the explicit assumption of the calibration mean, which is actually available. From the above discussion one sees that PG = SDR if the calibration and validation means are equal. On the other side, if the calibration and validation means are different, and if the model is performing adequately, then the predictions will distribute around mean($y_{val}$), meaning that the numerator in PG will be larger than that of SDR and PG > SDR. Working out the mathematical details one understands that PG rewards the capacity of a model to comply with different calibration and validation means, while SDR does not. This is why PG is a more fair statistic than SDR.

#### 2.6.1. PLS

**Figure 6:**
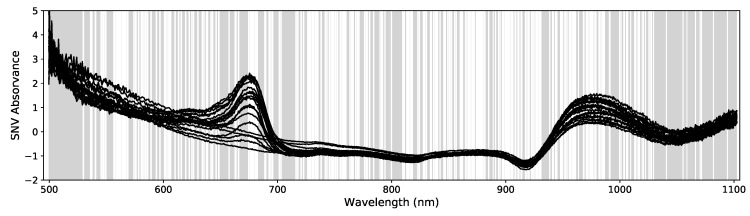
Example of 20 SNV absorbance spectra (abs_snv) taken from the *Big* data set. The gray shaded areas mark the wavelengths excluded by the PLS feature selection pre-processing (in this case 581). Only the remaining wavelengths are used for model optimizations.

In general, one of the most popular multivariate models for spectral analysis in Chemometrics is the Partial Least Square regression [36]. In our case we used the PLSRegression() function (which features an implementation of the NIPALS algorithm) available in the python SciKit-Learn package. In order to decrease the number of collinear variables in the spectra (neighbour wavelengths that vary the same way), we applied a ‘wrapper method’ to perform PLS feature selection to all data sets. Wrapper methods involve iteratively filtering features and refitting the model to optimize parameters. This kind of procedure has been shown to improve the model’s predictability by removing redundant and noisy features from the data (see review by [37]). We implemented an iterative method that consists in a double loop over features and latent variables [38] (Details available at https://www.idtools.com.au/variable-selection-method-pls-python/). In the first loop we apply the PLS model to all features, changing the number of latent variables (#LV) in the model until a predefined maximum. At each step we use 5 fold cross-validation and compute the RMSECV. The wavelengths are then sorted by importance of the PLS regression coefficient. In the second (inner) loop we iteratively remove one wavelength at a time (starting with the one with the lowest absolute value of the regression coefficient), refit the model and recompute the RMSECV. These nested loops compute the error for all the combinations of #LV and feature’s number. In the end, the procedure returns the features and the #LV in the PLS model that minimize the RMSECV for the calibration sets. This method is more computationally costly then using a simple PLS regression coefficient threshold but avoids the problems associated with the ambiguity/sensitivity of choosing that threshold in the first place. On the other hand, since we eliminate many collinear features, the posteriori application of the model becomes faster. After the best combination of #LV and discarded wavelengths is found for a specific calibration data set, we apply the PLS once more to that calibration set in 10k cross-validation and compute RMSEC. We believe that in this way the RMSEC is a more representative/trustworthy value of the calibration RMSE. Finally we also apply the PLS with the optimized #LV to the validation sets (pre-processed with the PLS feature selection) to compute the RMSEP. Table 1 and Figure 6 illustrate the result of this procedure for multiple data types (For simplification purposes, to describe each data type (variable) we use the following nomenclature: abs (absorbance), 1d (1st derivative), 2d (2nd derivative), _snv (standard normal variate) and the variables ended in 2 are those that include temperature and size appended in the *X-block*. For example abs2d2 corresponds to the absorbance (abs) 2nd derivative (2d) extended with temperature and size as additional features (2).).

**Figure 7:**
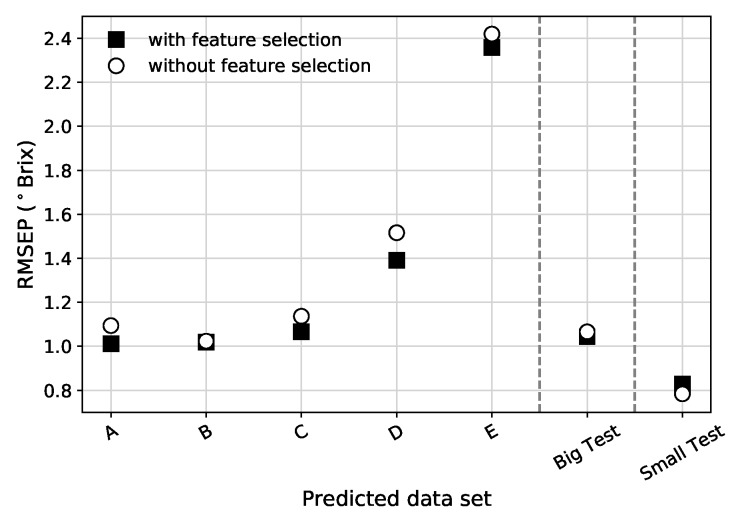
Comparison between RMSEP (in ${}^{\circ }$Brix) for all absorbance data sets, using the full spectrum, when using (or not using) the PLS feature selection procedure. The vertical dashed lines separate the different validation strategies.

For the vast majority of pre-processed data types and data sets, the PLS feature selection reduces the RMSEP (see Figure 7 for an example). PLS feature selection proved to improve not only the PLS model metrics but also the other tested models. Based on this, all the results presented from this point onward are based on data sets that were pre-processed with PLS feature selection.

#### 2.6.2. MLR

The second calibration model we studied was obtained by Multiple Linear Regression (MLR), which is one of the most simple techniques in multivariate analysis [8]. While PLS tries to maximize the covariance between the *X* and *Y* blocks, MLR looks to achieve maximum correlation between these two. As simple as it is, it has some drawbacks that limit the scope of its application in spectral analysis. For example in MLR, the number of features must be smaller than the number of samples otherwise the regression coefficients can not be computed reliably leading to over-fitting. This is precisely the case for our *Small* data set whose calibration subset only has 240 samples but a higher number of features (even after the PLS feature selection discarded many wavelengths). One alternative would be to decrease the number of features by applying a dimensionality reduction method such as Principal Component Analysis (PCA) or Singular Value Decomposition (SVD). Another possibility would be to choose a small number of wavelengths (for example those that correlate better with SSC) and create a series of spectral indices. This would decrease greatly the number of features (*X-block*) enabling the possible application of MLR to the *Small* set. However we want to maintain the integrity of the designed analysis pipeline as much as possible and for this reason, we didn’t pursued any of these alternatives in this work. Therefore we opted to apply the MLR model only to the *EV* and *Big* data sets.

#### 2.6.3. SVM

Support Vector Machines for regression, initially proposed by [39,40], has been used in recent years with great success in data analysis and is considered a very versatile technique in machine learning. We performed a series of preliminary tests where we applied the SVM regression model with different types of kernels to the *Big* calibration subset. From all the available options, the Radial Basis Function (RBF) kernel provided the best results. The efficiency of the SVM model is highly dependent of its hyper-parameters optimization, which means that in this case (RBF-kernel), hyper-parameters C, γ and ε have to be optimized (for each data set individually). C is the regularization parameter and it controls a tradeoff between the allowed error deviations and the complexity of the decision function, ε is related to the error penalty of the loss function and γ represents the width of the RBF kernel. Several optimization strategies have been suggested [41] and many involve hyper-parameter fine-tuning using computationally exhaustive grid-search and cross-validation approaches. However, the grid-search technique despite its high accuracy results, take a prohibitive computation time to achieve reliable results specially for schemes where re-configurations of the SVM model are required due to changes in the input calibration sets. This is exactly the case of our benchmark.

We adopted the hyper-parameter optimization proposed by [42]. This method uses a combination of theoretical hyper-parameter determination [43] coupled with a ‘restrained’ grid-search approach. We compared this method to an exhaustive grid-search using our *Big* calibration subset, and got similar results in a fraction of the time. In their work, the authors show that a good estimate of parameter C can be obtained analytically from the statistical distribution of the calibration data and, parameter ε can be obtained directly from a noise estimation in the data based on the mean squared error of a k Nearest Neighbors regressor algorithm. The theoretical value of γ is determined by maximizing the variance of kernel similarity diversity as detailed in [44]. However, these theoretical parameters, P${}_{th,i}$, are not optimized for prediction because of the statistical fluctuations that can exist in different sections of the calibration data. Fine-tuned values can be found in an interval around these theoretical values. Therefore we defined hyper-parameter uniform distributions between 0.001 × P${}_{th,i}$ and 3 × P${}_{th,i}$ and perform a randomized grid-search (RGS) for 30 candidates in these intervals. The optimization process takes two hyper-parameters (e.g., C and γ) as constant (equal to their theoretical values) and optimizes the 3rd one (e.g., ε) by RGS and minimization of RMSE using 5k cross-validation. This is done 3 times optimizing all hyper-parameters. Figure 2 of [42] illustrates the process. We found that for this method to work reliably the input data should lie in the [−1,1] range. To account for this restriction, we normalized our input data variables accordingly. Absorbance values were dived by 10, 1st derivative values were multiplied by 100 and 2nd derivatives were multiplied by 1000. Temperature and size data were mean centered and divided by 100. With these scalings all input features lie on the [−0.2,0.2] interval. The RMSEC presented is the value obtained by 5k cross-validation at the end of the last optimization step. The final RMSEP is computed by applying the SVM model with the optimized hyper-parameters to the validation data sets.

#### 2.6.4. MLP (NN)

For the Neural Network (NN) model we choose to use a Multi-Layer Perceptron (MLP) architecture with one hidden layer. All the data sets were standardized before feeding them to the NN. Besides this being an ‘advised good practice’ in the field, it was also necessary to uniformize data sets that include fruit size and temperatures as features (initially in different scales than the spectral features). We extracted the metrics used to standardize the calibration sets (i.e., $\langle x_{cal,i}\rangle$ and $std(x_{cal,i})$) and used them to subsequently standardize the validation samples. We also found that normalizing the *Y-block* increased the stability of the NN training. Afterwards, the predicted SSC values ($y_{pred,i}$) are rescaled back to the original non-standardized SSC scale to ensure that the computed error metrics are coherent with the other models.

For the used MLP, the number of neurons in the input layer was set equal to the number of features in the input data set, and since we are using this model for regression, the output layer consisted in one neuron. The number of neurons in the hidden layer was set to ‘1/3 of the number of input features’. We compared several thumb-rules to choose the number of neurons in the hidden layer as mentioned in [45] using our *Big* data set, and this ‘1/3 rule’ showed similar results in cross-validation when compared to a grid-search optimization. Moreover, since one of the objectives is to build a broad analysis pipeline that can be applied to multiple data sets, we wanted to avoid individual grid-search based fine-tuning for each data set. The optimization of the other NN parameters was also based in the *Big* data set. The NN training uses cross-validation (with an internal test split of 20% of the input calibration set), the ‘Adam’ optimizer, MSE as the loss function and a rectified linear unit (ReLU) as the activation function in the hidden layer. The ‘Adam’ optimizer is a more efficient implementation of the classical stochastic gradient descent optimization algorithm and allows for an iterative update of the neural network weights during the training phase [46]. We also implemented an early stop procedure that stopped the training when the MSE on the internal test split stopped improving. We defined the ‘Adam’ hyper-parameter ‘batch number’ as 16 samples and the maximum number of training epochs to 300. However, because of the early stop procedure, no data set training reached beyond 250 epochs. The weights for the best model were saved for posterior use and the predictions metrics were computed by applying the best trained model to the validation sets. Since the initialization weights of the NN are random and the calibration data is shuffled before the internal test split, this has as consequence that two fits to the same data set returns slightly different results. Therefore in order to have a much more consistent set of calibration/validation metrics, we use the average of 10 models fits to the same data set. The ability of running these models on a GPU provided very valuable in terms of the required computation time.

## 3. Results and Discussion

In order to facilitate the visualization of the results obtained with the 4 models, we display RMSEP and RMSEC for the multiple data sets in graphics and summarized the best prediction metrics for each data set into individual tables. Over the next paragraphs we highlight the most relevant results.

### 3.1. PLS and MLR Results

**Figure 8:**
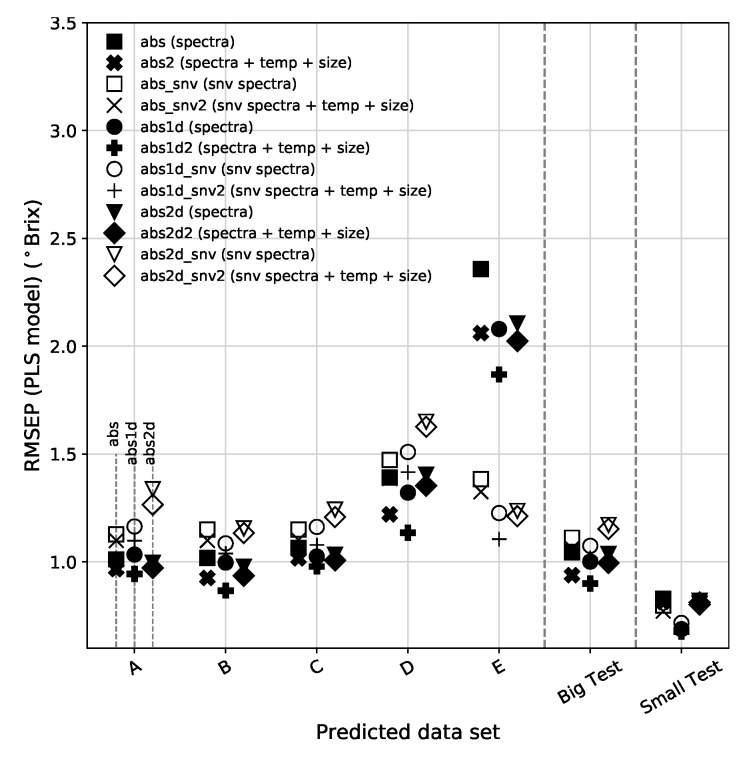

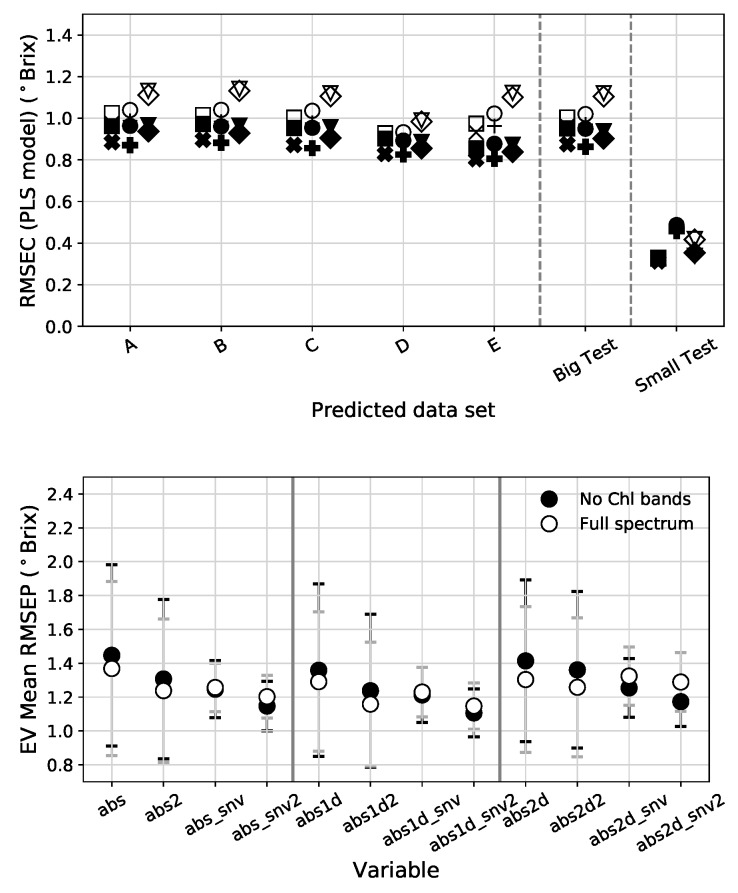
(**Top**): RMSEP for all validation sets using the full spectrum for the PLS model. For simplification purposes we are using the following nomenclature in the legends: abs (absorbance), 1d (1st derivative), 2d (2nd derivative), _snv (standard normal variate) and the variables ended in 2 are those that include temperature and fruit size. (**Middle**): RMSEC for PLS. (**Bottom**): Mean RMSEP for the *EV* sets, for all pre-processed variables, obtained using PLS. Results obtained using full spectra are represented by white circles (gray error bars) and those obtained excluding the Chl bands are plotted by black circles (black error bars). The vertical lines separate the sets based on absorbance, absorbance 1st derivative and absorbance 2nd derivative data sets. The error bars indicate the standard deviation of the RMSEP from all 5 *EV* predicted subsets.

From Figure 8 we can see that, in general, the SNV correction to the spectra (open/white symbols) tends to have slightly higher RMSEP than their original counterparts, with the exception of data set E (and to a lesser extent the *Small* data set). In order to understand the success of SNV in bringing the predictions of subset E down to acceptable values, it is important to realize that this subset has a large proportion of small and large fruit sizes, and that the largest errors of prediction are found in these sizes. In our optical setup there are significant changes in the distance between incidence and collection spots for both small and large pears, which in turn induce changes in the spectra. However, the SNV transformation is effective in compensating these changes. On the other side, the derivative transformation, which is usually assumed to be as efficient or better than SNV to compensate multiplicative scattering effects, is not so effective in this case (only derivative + SNV is effective). One other characteristic is that the data types that get the lowest RMSEP are those that include the temperature and fruit size as extra features, in particular those based on the absorbance 1st derivative.

The bottom panel of Figure 8 shows a comparison of the mean RMSEP for the 12 pre-processed data types using the full spectra and excluding the Chl bands. Table 2 summarizes the most relevant results. We find that the best overall result, although by a very small margin, for the PLS model comes by computing the absorbance 1st derivative of the spectra without Chl bands, corrected by SNV and extended with temperature and fruit size information. The global result is a slight improvement on SNV-treated models with the addition of the two extra variables and a slight degradation on the other models. This is probably related to the fact that the SNV correction to the spectra without Chl produces a much more regular/uniform set of spectra than that of the SNV applied to the full spectra. The variabilities associated with the Chl peak at 680 nm and the beginning of the spectra at 500 nm decrease the effectiveness of the SNV correction.

The results based on the *Big* and *Small* sets indicate a higher predictability of the PLS using the absorbance 1st derivative extended with temperature and fruit size. For these two data sets, the full spectra version, provides the best results. Although the RMSEP of the *Small* data set is lower than that of the *Big* data set, the Prediction Gain from the latter is much more significant. This is justified by the fact that the range of SSC available in the *Small* set is much narrower than that of the *Big* set.

The RMSEP and RMSEC obtained using the MLR model show a similar pattern to that found in Figure 8 for the PLS model with just a couple os slight increases here and there. Table 3 shows the summary of the calibration and prediction metrics for the *EV* set and *Big* set.

We also would like to notice that a comparison of the results obtained with PLS and MLR is useful to understand if the PLS feature selection pre-processing is good enough to decrease the collinearity in the data (collinearity degrades the MLR performance). We tested this by applying the MLR model to the full 1024 features and we could see that the results were worst. Another useful information that sprouts from this comparison is the ability to infer if the relationship between spectra and SSC could be interpreted as linear. The similarity in the performance between both models for the *EV* and *Big* data set is an indicative that this true. PLS pulls ahead in terms of performance but by a very small margin that, for most practical applications, can be considered statistically irrelevant.

### 3.2. SVM and MLP Results

**Figure 9:**
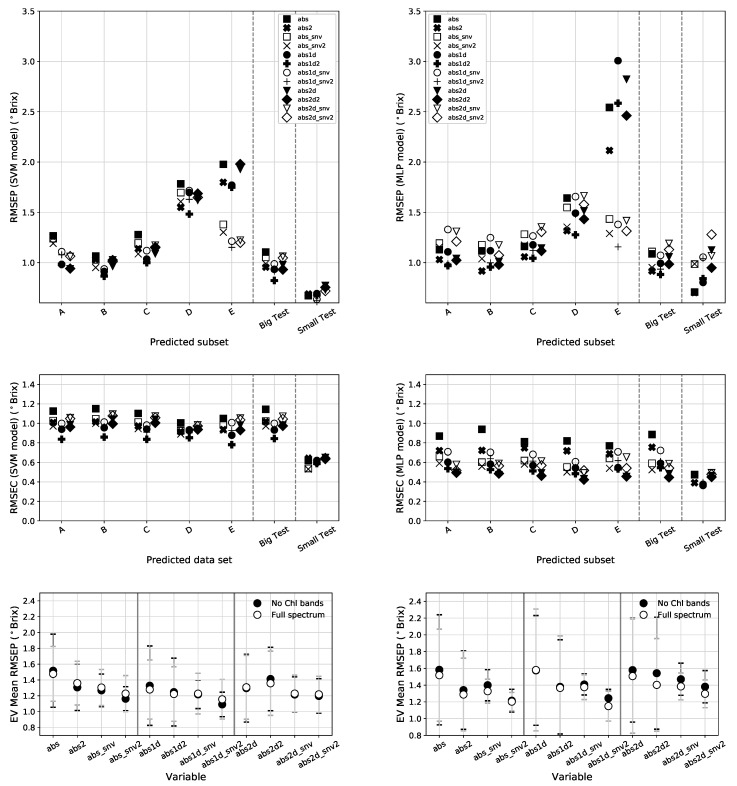
Top: RMSEP for all validation sets (using the full spectral range) for the SVM regression model (**left**) and the MLP NN (**right**). The mid panels shows the RMSEC for these models and the bottom panels display a comparison between the EV mean RMSEP obtained using the full spectral range and by excluding the Chl bands.

Table 4 and the left panels of Figure 9 shows the error metrics found using the SVM model. By looking at the RMSEC panel, we can see that this model fits all types of data almost the same way in terms of calibration, with slight better results for the absorbance 1st derivative extended with temperature and fruit size. The RMSEP panel shows a prediction pattern that resembles that of the PLS model. For the *EV* subsets, D and E present the largest errors with the SNV version of E showing better performance. The *Big* and *Small* data sets maintain the calibration integrity that can be seen by the close RMSEC and RMSEP values. This calibration model shows the best *EV* metrics of the benchmark with an RMSEP of 1.09 ${}^{\circ }$Brix found using the absorbance 1st derivative extended with temperature and fruit size on the spectra without the Chl bands. This is a marginal improvement over PLS for the same variable. The SVM also provides the best metrics for the *IVBig* and *Small* sets with RMSEPs of 0.82 ${}^{\circ }$Brix (on abs1d2) and 0.62 ${}^{\circ }$Brix (on abs1d_snv2) respectively.

Figure 9 presents in the right panels the error metrics for the MLP NN model and Table 5 shows the summary of the best data types. For this model, the best preprocessed data type for the *Big* set is the absorbance 1st derivative augmented with temperature and fruit size information using the full spectral range. As for the *EV* set, the results show an increased RMSEP of 1.15 obtained using the SNV corrected absorbance 1st derivative augmented with temperature and fruit size. The *Small* set, shows an RMSEP of 0.70 but since the brix range values is narrower than in the other sets, this translates in a worse PG. The prediction made using the MLP shows the same general pattern obtained for the previous calibration models. Nevertheless the difference between RMSECs and RMSEPs hint that this MLP model might be suffering from some degree of overfitting or could be better optimized.

### 3.3. Discussion and Remarks

From the 3 predefined validation strategies, the lowest prediction errors were obtained using the *Small* set with the SVM calibration model (RMSEP = 0.62 ${}^{\circ }$Brix). This is was somewhat expected for the reasons pointed out in the introduction, i.e., it is a much more uniform data set and the SSC distribution is narrower than in the others. A quantitative comparison with other works is hard because there is no similar data about ‘Rocha’ pear published, and even for different pear cultivars, authors used different calibration models, data pre-processing techniques and spectral ranges. That being said, we can attempt a qualitative comparison. Our results obtained with the PLS model (RMSEP = 0.68 ${}^{\circ }$Brix) are on par with some of the results present in the literature based on this same type of model. Using PLS applied to spectral data sampled over a broad wavelength range (350–1800 nm), ref. [12] reported an RMSEP = 0.66 ${}^{\circ }$Brix for a set of 80 ‘Fengshui’ pears. Other authors that implemented more sophisticated pre-processing/modelling techniques achieved better metrics. For example recently, ref. [14] reported an RMSEP ≈ 0.48 ${}^{\circ }$Brix using PLS on 240 ‘European’ pears and pre-processing the spectra using Orthogonal Signal Correction; ref. [24] reported an RMSEP = 0.526 ${}^{\circ }$Brix also using PLS on a set of 120 ‘Korla’ pears that included a feature selection pre-processing using a genetic algorithm named Competitive Adaptive Reweighted Sampling developed by [47]. Furthermore, besides PLS, SVM has also been used and showed great potential. In [26], the authors used a combination of Monte-Carlo-Uninformative variable elimination, Successive Projections Algorithm and Least Squares SVM on 240 pears (from 3 different varieties) to achieve an RMSEP as low as 0.32 ${}^{\circ }$Brix. Although these results that are obtained using more advanced analysis techniques seem promising, at this point we cannot be sure if the merit of the achievement is due to algorithmic improvement or to the small sets size and their statistical homogeneity.

For the *Internal Validation* strategy (*Big* set) most models reported an RMSEP below ∼0.9 ${}^{\circ }$Brix with the exception of the MRL model that fairs a bit worse at 0.91 ${}^{\circ }$Brix. Overall, the SVM calibration model provides the best metrics followed by the MLP. Using *IV*, all models report the best predictions based on the absorbance 1st derivative extended with temperature and size information. This is also where PG and R${}^{2}$ are higher. A comparison between RMSEC and RMSEP values for the SVM, PLS and MLR models indicate that these models are not overfitting. In contrast, looking at Table 5 and Figure 9, we can see clear differences between RMSEC and RMSEP obtained using the MLP. This difference hints that the MLP calibration model is probably suffering from a slight overfitting on the *Big* data set for most of the data types. A thorough/computationally expensive grid-search or a bayesian hyper-parameter optimization could potentially lead to improved results in this scenario of *Internal Validation*. Our MLP results are similar to those reported by [16] that used a more complex NN architecture, an Extreme Learning Machine and Successive Projections Algorithm pre-processing on 100 pears (Zaosu and Huangguan varieties) to achieve a RMSEP = 0.89 ${}^{\circ }$Brix. We view our results as an opportunity for future model improvement because we have a much higher volume of data, we can test different optimization strategies for different data types and we can probe more sophisticated NN architectures. For example, Convolutional Neural Networks (CNNs) have been recently used in the VIS-NIR spectroscopy area for compound quantification with interesting results [48,49,50].

As mentioned earlier we believe that the *External Validation* provides a more robust way to measure how a model would behave if deployed in the real world. In this case, for the *EV* data set, the best results were obtained using the SVM model, on the SNV corrected absorbance 1st derivative extended with temperature and fruit size (abs1d_snv2) using the spectral range that excludes the Chl bands, with a RMSEP = 1.09 ${}^{\circ }$Brix. The PLS and MLR models provided very similar results. When we look at the prediction of individual *EV* subsets (A, B, C, D, E) we find that for most subsets the SNV correction gives higher RMSEP than their non-SNV counterparts. However, when a systematic bias appears in some chunk of the data (of experimental or biological origin), SNV correction provides a major advantage. We see this in the prediction of *EV* subset E (see Figure 8). In this specific case, the training data (ABCD) is largely derived from average size fruits while the first half of E is basically constituted by average sizes and the second half by big and small fruits (where the prediction error is higher). As we explained earlier, SNV alleviates this discrepancy. Another factor that could be influencing the higher prediction errors found for subsets D and E is the harvest season. The prediction of D and E are based on calibrations made with 3 subsets from the 2010 harvest (ABC) and only 1 from the 2011 harvest (D or E). This supports the empirical knowledge that fruit’s chemistry change from season to season.

The development of model optimization methods, based on heterogeneous data sets would also be advantageous. Moreover, according to some of the cited literature, the use of a Least Squares SVM variant and NN architectures recently developed for deep-learning could lead to better results. The downside of this last remark is that the SVM and NN optimizations are very complex (and some times tricky) to implement. Their hyper-parameters space is large and their optimization methods depend heavily on the behaviour of data. Sometimes, a slight rescaling of the data can have big impacts on these models accuracy. On the other hand, PLS and MLR can be easily optimized providing results on par of the more complex models. This tradeoff is perhaps one of the main reasons for the large base of adoption of PLS.

## 4. Conclusions

Our experiment shows the feasibility of implementation of an affordable optical module, based on non-contact VIS-NIR reflectance spectroscopy, for non-destructive SSC quantification in ‘Rocha’ pears, in real time in existing sorting facilities. The prediction error of such a system should be around 1 ${}^{\circ }$Brix for individual fruit classification. The different results obtained using *Internal Validation* and *External Validation* strategies indicate that further optimizations are required in order to go below that threshold. We advocate that *External Validation* methods give more reliable results for this kind of problem because of the inherent temporal axis present in the data. In the case of fruit characterization, the validation data (e.g., next harvest) is always disconnect from the calibration data (previous harvests). Moreover, we believe that the non uniform SSC distribution inside fruits and the measurement error associated with SSC laboratory destructive tests done to generate the data, establish a lower limit for model predictions. Given the SSC variability measured in each fruit individually, at this point we believe that an RMSEP below 0.5 ${}^{\circ }$Brix for inline fruit calibration of ‘Rocha’ pear in real time is very unlikely. The ∼1 ${}^{\circ }$Brix error that can be obtained using this methodology will provide much better statistical assessment in terms of fruit batches than the destructive methods currently in place in fruit packinghouses. Besides, there is still room for some improvements in terms of modelling. This is already a significant improvement to the standard procedure, because it is fast, non-destructive and assess all and each fruit on the automated inline fruit sorting system, with a minor deviation from the error resulting from the standard destructive refractometer method currently used.

In terms of modelling, the best overall results for *EV* are obtained using SVM applied to the spectra excluding the Chl bands using the absorbance 1st derivative corrected by SNV and extended with temperature and fruit size information. PLS also showed good prediction potential with the advantage of having a simpler implementation and optimization procedure than the SVM. From a statistical point of view, a t-test between the predictions errors obtained with both models shows no significant difference. The use of additional information of temperature and fruit size improved the results along all models. This is a sign that multivariate models can capture and separate the information due to chemical and temperature spectral changes. Since many sorting facilities already include size and temperature sensors it will be easier to combine this extra information with the spectral information to improve SSC predictions. Even in sorting facilities were temperature sensors are not used, in principle it is possible to extract temperature information directly from the spectral data and perform a reconstruction of the temperature profile. This is possible because there is a very high correlation and linear relation between temperature and some spectral features (Cavaco et al. unpublished).

Although in this work we focus was the characterization of ‘Rocha’ pear, the general principles and methodology presented can be extended and optimized to other types of thin peel fruits.

## Figures and Tables

**Table 1:** Number of wavelengths discarded and optimal #LV determined (in parenthesis) by the PLS feature selection pre-processing for all data types and calibration (train) sets using the full spectrum (500–1100 nm) and excluding the Chl bands (730–1100 nm). Data types that include temperature and size use the same corresponding spectral features as these.

	abs	abs_snv	abs1d	abs1d_snv	abs2d	abs2d_snv
ABCD (full spec.)	419 (19)	575 (17)	542 (22)	728 (25)	585 (25)	608 (25)
ABCE (full spec.)	439 (18)	536 (17)	637 (25)	698 (25)	534 (25)	587 (24)
ABDE (full spec.)	472 (18)	604 (18)	540 (25)	692 (25)	633 (24)	650 (22)
ACDE (full spec.)	459 (18)	523 (23)	576 (25)	578 (25)	598 (25)	597 (25)
BCDE (full spec.)	489 (17)	528 (18)	598 (23)	581 (24)	593 (19)	552 (25)
Big train (full spec.)	413 (19)	581 (19)	595 (25)	622 (25)	623 (22)	612 (23)
Small train (full spec.)	579 (22)	543 (23)	560 (23)	798 (13)	651 (23)	691 (24)
ABCD (no Chl)	311 (13)	309 (11)	430 (25)	378 (24)	328 (22)	429 (24)
ABCE (no Chl)	263 (12)	333 (9)	438 (25)	418 (24)	341 (25)	417 (24)
ABDE (no Chl)	245 (13)	365 (10)	384 (24)	374 (25)	356 (24)	381 (24)
ACDE (no Chl)	256 (13)	289 (10)	458 (22)	427 (25)	356 (25)	414 (18)
BCDE (no Chl)	227 (14)	306 (10)	410 (24)	399 (24)	348 (25)	373 (22)
Big train(no Chl)	250 (13)	276 (14)	406 (23)	423 (25)	374 (24)	393 (23)
Small train (no Chl)	310 (18)	263 (18)	455 (25)	418 (25)	349 (25)	386 (25)

**Table 2:** Prediction metrics for PLS calibration model (Best pre-processing, RMSEC, RMSEP, R${}^{2}$, % and PG). EV${}^{*}$ values refer to the mean of all EV subsets.

	Best Preproc.	RMSEC	RMSEP	R${}^{2}$	%	PG
EV${}^{*}$ (full spec.)	abs1d_snv2	0.96	1.15	0.56	8.86	1.55
Big (full spec.)	abs1d2	0.86	0.90	0.73	6.91	1.91
Small (full spec.)	abs1d2	0.46	0.68	0.62	4.95	1.57
EV${}^{*}$ (no Chl)	abs1d_snv2	0.97	1.11	0.58	8.57	1.60
Big (no Chl)	abs1d2	0.90	0.98	0.68	7.54	1.75
Small (no Chl)	abs1d2	0.48	0.76	0.55	5.60	1.38

**Table 3:** Prediction metrics for MLR model (Best pre-processing, RMSEC, RMSEP, R${}^{2}$, % and PG). EV${}^{*}$ values refer to the mean of all EV subsets.

	Best Preproc.	RMSEC	RMSEP	R${}^{2}$	%	PG
EV${}^{*}$ (full spec.)	abs1d_snv2	0.89	1.15	0.55	8.9	1.54
Big (full spec.)	abs1d2	0.78	0.91	0.72	7.0	1.88
EV${}^{*}$ (no Chl)	abs1d_snv2	0.91	1.11	0.57	8.63	1.59
Big (no Chl)	abs1d2	0.85	0.99	0.67	7.56	1.74

**Table 4:** Prediction metrics for SVM model. EV${}^{*}$ values refer to the mean of all EV subsets.

	Best Preproc.	RMSEC	RMSEP	R${}^{2}$	%	PG
EV${}^{*}$ (full spec.)	abs1d_snv2	0.93	1.16	0.57	8.94	1.56
Big (full spec.)	abs1d2	0.84	0.82	0.77	6.31	2.09
Small (full spec.)	abs1d_snv2	0.59	0.62	0.66	4.59	1.71
EV${}^{*}$ (no Chl)	abs1d_snv2	0.92	1.09	0.60	8.45	1.63
Big (no Chl)	abs1d2	0.84	0.82	0.77	6.33	2.09
Small (no Chl)	abs1d	0.63	0.63	0.68	4.56	1.70

**Table 5:** Prediction metrics for MLP (NN) model. *EV*${}^{*}$ values refer to the mean of the 5 *EV* subsets. All values are an average of the metrics over 10 models.

	Best Preproc.	RMSEC	RMSEP	R${}^{2}$	%	PG
EV${}^{*}$ (full spec.)	abs1d_snv2	0.60	1.15	0.57	8.89	1.55
Big (full spec.)	abs1d2	0.54	0.88	0.75	6.78	1.96
Small (full spec.)	abs2	0.39	0.70	0.58	5.15	1.53
EV${}^{*}$ (no Chl)	abs_snv2	0.49	1.21	0.51	9.64	1.46
Big (no Chl)	abs1d2	0.58	0.92	0.73	7.09	1.86
Small (no Chl)	abs	0.49	0.75	0.51	5.52	1.41

## Abbreviations

The following abbreviations are used in this manuscript:

SSC	Soluble Solids Content
VIS-SWNIR	Visible and Short Wave Near Infrared
SNV	Standard Normal Variate
MSC	Multiplicative Scatter Correction
Chl	Chlorophyll
PLS	Partial Least Squares
#LV	Number of Latent Variable (PLS model)
SVM	Support Vector Machines
MLR	Multiple Linear Regressions
MLP	Multi-Layer Perceptron
NN	Neural Network
RGS	Randomized Grid Search
GPU	Graphics Processing Unit
IV	Internal Validation
EV	External Validation
RMSEP	Root Mean Squared Error of Prediction
RMSEC	Root Mean Squared Error of Calibration
RMSECV	Root Mean Squared Error of Calibration using Cross-Validation
R${}^{2}$	Coefficient of Determination
PG	Prediction Gain
SDR	Standard Deviation Ratio
